# Survival benefit of IABP in pre- versus post-primary percutaneous coronary intervention in patients with cardiogenic shock

**DOI:** 10.1186/s43044-024-00527-w

**Published:** 2024-08-06

**Authors:** Ahmed Azazy, Walaa Abdaziz Farid, Walid Abdu Ibrahim, Wassam ELDin Hadad El Shafey

**Affiliations:** 1 Department of Cardiology, King Saud Medical City, Riyadh, Saudi Arabia; 2 Department of Cardiology, Menofiya University Hospital, Shebin El Kom, Egypt

**Keywords:** Acute myocardial infarction, Cardiogenic shock, Intra-aortic balloon counter-pulsation, Mortality, Percutaneous coronary intervention

## Abstract

**Background:**

Cardiogenic shock (CS) remains a major cause of in-hospital mortality in the setting of acute myocardial infarction (AMI). However, little evidence is available regarding the optimal order of intra-aortic balloon counter-pulsation (IABP) insertion and primary percutaneous coronary intervention (PPCI). The aim of this study was to assess the hospital and short-term survival benefits of two different IABP insertion approaches, before versus after PPCI in patients with acute myocardial infarction and cardiogenic shock.

**Results:**

Total mortality was 80 patients representing 48.4% of the total 165 studied patients; 60 patients died during the hospital admission period, while the remaining 20 patients died post-discharge. In-hospital mortality was significantly higher in Post-PPCI–IABP group 40 (49.4%) versus Pre-PPCI–IABP group 20 (23.8%) (*P* = 0.001). Moreover, the mortality difference between the two groups was sustained over six-month follow-up period, where 15 patients (18.5%) died in the Post-PPCI–IABP group, while only 5 patients 6.0% died in the Pre-PPCI–IABP (*P *= 0.001).

**Conclusions:**

Early IABP insertion before PPCI is associated with improved in-hospital and long-term survival when used for patients presenting with AMI complicated by hemodynamic instability.

## Background

Intra-aortic balloon counter-pulsation (IABP) has been increasingly used as a mechanical circulatory support in cardiogenic shock (CS) patients with post-acute myocardial infarction (AMI) by providing circulatory assistance to the failing left ventricle (LV) [1]. Diastolic inflation and systolic deflation of the counter-pulsation balloon generate kinetic energy in the aortic root that results in LV after-load and cardiac work reduction and thus leads to decrease in myocardial oxygen demand. Moreover, the augmented effect of IABP on diastolic pressure is the increase in coronary perfusion and reduction of LV filling pressure [2]. The reduction of ischemic burden and infarct size post-acute AMI is the net effect of IABP [3]. In the USA, the use of IABP in AMI accounts for one-third of the cardiovascular procedures [4]. The hemodynamic support after cardiac catheterization is the common indication of IABP placement in 20%, followed by CS in 19%, cardiopulmonary bypass weaning in 16%, preoperative use in 13%, and refractory unstable angina in 12% as shown by The Benchmark Registry [5]. Until now, there are no obvious recommendations about the IABP optimal duration post-ST-elevation myocardial infarctions (STEMI) although the use of IABP for more than two days results in a significant increase in vascular complications, gastrointestinal bleeding, and infection [6].

Various clinical randomized-controlled trials demonstrated a better prognosis and short-term survival with simultaneous use of primary percutaneous coronary intervention (PPCI) with IABP in AMI [7–9]. However, a few randomized trials and recent meta-analyses showed that IABP insertion after PPCI did not improve in-hospital and short-term survival [10, 11].

In this study, we analyzed the in-hospital and short-term survival benefits of two different IABP insertion approaches, before versus after PPCI in patients with STEMI complicated by CS.

## Methods

This study is a single-center non-randomized retrospective trial that aims to assess the hospital and short-term survival benefits of two different IABP insertion approaches, before versus after PPCI in patients with acute myocardial infarction and cardiogenic shock. We retrospectively reviewed the data of 165 patients with hemodynamically unstable acute coronary syndrome treated with IABP insertion and PPCI in King Saud Medical City, a tertiary care hospital between January 2017 to February 2022. Patients' baseline characteristics including demographics, clinical presentation, and procedural and post-procedural complications were collected. The study protocol was approved by the regional ethical committee. The decision and timing of IABP insertion were based mainly on the clinical situation, international guidelines, and operator preferences. All IABPs were inserted under fluoroscopy guidance in the catheterization laboratory using 8-French catheters (Arrow Corp, Reading, PA, USA). Patients referred for emergency or urgent coronary artery bypass graft surgery were excluded from the study. The patients were divided into two groups according to IABP insertion time: The first group, (*n* = 84 patients) had counter-pulsation support started before PPCI (Pre-PPCI–IABP group) and the second group (*n* = 81patients) who had the counter-pulsation support started after PPCI (Post-PPCI–IABP group).

Diagnosis of STEMI and non-STEMI was based on symptoms and the European Society of Cardiology electrocardiogram criteria [12, 13]. CS was defined as persistent systolic blood pressure of < 90 mmHg, uses of vasopressors to maintain the systolic blood pressure of > 90 mmHg and evidence of end-organ hypo-perfusion such as altered mental status, oliguria or cold extremities that does not respond to fluid resuscitation [8]. Door-to-balloon time was defined as the interval between the hospital's arrival and the first balloon dilatation of the proposed culprit artery. If required, vasopressors (dopamine, norepinephrine and/or epinephrine) were used and tittered with continuous hemodynamic monitoring. Dual anti-platelets, anticoagulation, and glycoprotein IIb/IIIa were used according to the European Society of Cardiology guidelines [12, 13]. The main outcome was in-hospital mortality and short-term (6 months) mortality.

The data were analyzed using SPSS IBM., Chicago, IL. Continuous normally distributed variables were represented as mean ± SD. with 95% confidence interval, while non-normal variables were summarized as median with 25 and 75 percentiles, and using the frequencies and percentage for categorical variables; a *p* value < 0.05 will be considered statistically significant. To compare the means of normally distributed variables between groups, the Student’s t test was performed, the Mann–Whitney *U* test was used in non-normal variables and *χ*2 test or Fisher’s exact test was used to determine the distribution of categorical variables between groups. Spearman's rank correlation coefficient (*r*) was used to show the correlation between different parameters in this study. Logistic regression analysis was done to assess the risk factors for the death rate of the studied patients. Significant predictors in the univariate analysis were included in a stepwise forward multivariate analysis (*P* < 0.05 for entering the model and *P* < 0.1 for removal from the model) to determine the final predictor factors for death.

## Results

In total, 165 patients were recruited in this study. There were no significant differences between the two groups regarding age, body mass index, rate of smoking, diabetes, hypertension, hypercholesterolemia, blood pressure, and cardiac biomarkers. Patients with Post-PPCI–IABP have a significantly higher prevalence of prior MI history compared to Pre-PPCI–IABP (25/81 (30.9%) vs. 5/84 (6.0%), *P* = 0.001), respectively **(**Table 1**)**.

**Table 1:** Demographic, clinical, and investigations data

			IABP PRE-PPCI *N* = 84	IABP POST-PPCI *N* = 81	*P* value	OR (95%CI)	*P* value
Demographic data	Age		52.1 ± 10.5	50.0 ± 12.8	0.2	0.98 (0.96- 1.01)	0.2
	Sex	Female	10 (11.9%)	10 (12.3%)	0.9	0.96 (0.38- 2.44)	0.9
		Male	74 (88.1%)	71 (87.7%)			
	HTN	No	64 (76.2%)	51 (63.0%)	0.06	1.9 (0.96- 3.7)	0.06
		Yes	20 (23.8%)	30 (37.0%)			
	DM	No	30 (35.7%)	40 (49.4%)	0.07	0.57 (0.31- 1.06)	0.07
		Yes	54 (64.3%)	41 (50.6%)			
History	PCI	No	79 (94.0%)	56 (69.1%)	0.001**	7.06 (2.55- 19.55)	0.001**
		Yes	5 (6.0%)	25 (30.9%)			
	CABG	No	84 (100.0%)	81 (100.0%)	N.A	-	N.A
		Yes	0 (0.0%)	0 (0.0%)			
VITALS	SBP		102.2 ± 22.3	114.9 ± 22.4	0.001**	1.03(1.01- 1.04)	0.001**
	DBP		60.2 ± 20.9	68.0 ± 17.0	0.01*	1.02 (1.01—1.04)	0.01*
	HR		106.4 ± 31.6	97.9 ± 29.0	0.07	0.991 (0.98- 1.01)	0.07
	SpO2		91.9 ± 5.0	92.1 ± 6.0	0.8	1.01 (0.95- 1.06)	0.8
Risk Factor	DL	No	64 (76.2%)	56 (69.1%)	0.2	1.43 (0.72- 2.85)	0.3
		Yes	20 (23.8%)	25 (30.9%)			
	SK	No	59 (70.2%)	66 (81.5%)	0.06	0.54 (0.26- 1.1)	0.09
		Yes	25 (29.8%)	15 (18.5%)			
	CKD	No	74 (88.1%)	81 (100.0%)	0.001**	0.01 (0.001 – 0.24)	0.9
		Yes	10 (11.9%)	0 (0.0%)			
	PVD	No	84 (100.0%)	81 (100.0%)	N.A	-	N.A
		Yes	0 (0.0%)	0 (0.0%)			
	Old MI	No	79 (94.0%)	56 (69.1%)	0.001**	7.06 (2.55- 19.55)	0.001**
		Yes	5 (6.0%)	25 (30.9%)			
KILLIP CLASS		II	0 (0.0%)	5 (6.2%)	0.001**	-	-
		III	20 (23.8%)	45 (55.6%)		2.3 (1.15- 4.49)	0.001**
		IV	64 (76.2%)	31 (38.3%)		1.4 ( 0.78- 1.87)	0.001**
EF < 35%		> 35%	5 (6.0%)	35 (43.2%)	0.001**	12.02 (4.4- 32.8)	0.001**
		< 35%	79 (94.0%)	46 (56.8%)			
LABS	HB		13.5 ± 2.3	14.0 ± 2.9	0.2	1.08 (0.96—1.2)	0.2
	CREA		128.0 (107.0—208.0)	95.0 (65.0—127.5)	0.001**	0.982 (0.975- 0.990)	0.001**
	TROP		19.7 ± 18.3\	18.2 ± 18.0	0.6	0.99 (0.98- 1.01)	0.6
	CKMB		166.0 (42.0—420.0)	159.0 (80.8—382.0)	0.5	0.99 (0.99- 1.0)	0.03*
	PLT		279.5 ± 76.3	329.1 ± 158.2	0.01*	1.05 (1.01- 1.07)	0.02*
SYMPTOMS DURATION/hrs			6.0 (4.0—24.0)	5.0 (2.5—12.0)	0.01*	0.98 (0.96- 1.05)	0.13

Data are presented as *n* (%) or mean ± SD. *CKD* chronic kidney disease; *DM* diabetes mellitus; *HR*: heart rate; *HTN* Hypertension; *IABP* intra-aortic balloon pump; *MI* myocardial infarction; *NSTEMI* non ST elevation MI; *PVD* peripheral vascular disease; *PCI* percutaneous coronary intervention; *SpO*_*2*_ oxygen saturation; *STEMI* ST elevation MI

^*^P value < 0.05 is significant, **p value < 0.01 is highly significant

Almost all patients had symptomatic heart failure, 65/165(48.1%) with Killip class-III (acute pulmonary edema) and 70/165 (51.9%) with class-IV (cardiogenic shock). Killip class-IV and severely reduced left ventricle ejection fraction (LVEF) of  < 35% was more common in the Pre-PPCI–IABP than in the Post-PPCI–IABP group: 64/84 (76%) versus 31/81 (38%), *P* = 0.001; and 79/81 (94%) vs. 46/84 (56.8%), *P* = 0.001, respectively. The other clinical characteristics of the two groups are illustrated in Table 1.

STEMI was significantly higher in Post-PPCI–IABP [59/ 84 (70.2%) vs 71/ 81 (87.7%), *P* = 0.01], while left main coronary artery and/or multi-vessel disease and door-to-balloon time was significantly greater in the Pre-PPCI–IABP when compared to the post-PCI–IABP group: 20/84 (23.8%) versus 44/84 (52.4%), *P* = 0.04; and (74.3 ± 8.2 vs. 62.0 ± 7.8, *p* = 0.001), respectively. Complete revascularization was achieved successfully in the Post-PPCI–IABP when compared to the Pre-PPCI–IABP group 25/84 (30%) versus 15/81 (17%), *p* = 0.04 (Table 2).

**Table 2:** Procedural data of both groups

			IABP PRE-PPCI *N* = 84	IABP POST-PPCI *N* = 81	*P* value
TYPE	NSTEMI		25 (29.8%)	10 (12.3%)	0.01*
	STEMI		59 (70.2%)	71 (87.7%)	
VESSELS INVOLVED	LM	No	64 (76.2%)	71 (87.7%)	0.04*
		Yes	20 (23.8%)	10 (12.3%)	
	LAD	No	30 (35.7%)	25 (30.9%)	0.3
		Yes	54 (64.3%)	56 (69.1%)	
	LCX	No	69 (82.1%)	51 (63.0%)	0.01*
		Yes	15 (17.9%)	30 (37.0%)	
	RCA	No	49 (58.3%)	56 (69.1%)	0.1
		Yes	35 (41.7%)	25 (30.9%)	
	MVD	No	40 (47.6%)	50 (61.7%)	0.04*
		Yes	44 (52.4%)	31 (38.3%)	
COMPLETE REVASCULARIZATION		No	69 (82.1%)	56 (69.1%)	0.04*
		Yes	15 (17.9%)	25 (30.9%)	
Glycoprotein inhibitors IIb/IIIa		No	40 (47.6%)	15 (18.5%)	0.001**
		Yes	44 (52.4%)	66 (81.5%)	
USE OF VASOPRESSORS		No	25 (29.8%)	35 (43.2%)	0.05*
		Yes	59 (70.2%)	46 (56.8%)	
MECHANICAL VENTILATION		No	54 (64.3%)	61 (75.3%)	0.08
		Yes	30 (35.7%)	20 (24.7%)	
Door-to-balloon time/ minutes(mean ± SD)			74.3 ± 8.2	62.0 ± 7.8	0.001
DEATH RATE		Live	59 (70.2%)	26 (32.1%)	0.001**
		Death in Hospital	20 (23.8%)	40 (49.4%)	0.001**
		Death after 6 Months	5 (6.0%)	15 (18.5%)	0.001**

*LM* left main; *LAD *left anterior descending; *LCX* left circumflex; *RCA* right coronary artery

^*^P value < 0.05 is significant, **p value < 0.01 is highly significant

During this retrospective study, we did not elucidate any known complications of IABP insertion or early (before intervention) cardiac arrests in our database.

## In-hospital and short-term outcome

**Fig. 1:**
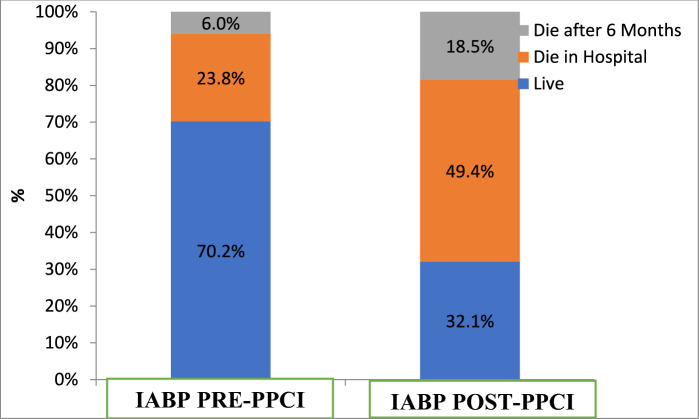
Death rate in the studied groups

In total, mortality was 80/165 (48.4%), of whom 60/165 (36.3%) died in-hospital and 20/165 (12.1%) died six months after discharge. The total and out-of-hospital death was significantly higher in the Post-PPCI–IABP group: 25/84 (29.8%) versus 55/81 (67.9%), *P* = 0.001, and 15/81 (18.5%) versus 5/84 (6%), *P* = 0.001, respectively, while in-hospital mortality was significantly higher in the Post-PPCI–IABP group versus the Pre-PPCI–IABP group: 40/81 (49%) versus 20/84 (23%), *P* = 0.001 (Table 2 and Fig. 1**)**.

CKMB and creatinine levels were significant predictors of death in the Pre-PPCI–IABP group with an adjusted odd ratio (AOR): 1.002, confidence interval (CI): 1.001–1.03, *P* = 0.002; and 1.028 (1.014–1.091), *P* = 0.001, respectively, while in Post-PPCI–IABP, LVEF of < 35% and creatinine level were the most significant predictors of poor outcome: AOR of 5.04, CI 1.66–15.29, *P* = 0.004 and 1.017 CI 1.004–1.029, *P* = 0.008, respectively **(**Table 3**)**.

**Table 3:** Risk assessment of the following predictor factors on the death rate regarding the studied groups

Adjusted risk factors	Death Rate in IABP PRE–PPCI				Death Rate in IABP POST–PPCI			
	AOR	95.0% C.I		*P* value	AOR	95.0% C.I		*P* value
		Lower bound	Upper bound			Lower bound	Upper bound	
RISK FACTORS_MI	0.733	0.234	2.297	0.6	1.001	0.987	1.023	0.9
KILLIP CLASS	1.003	0.969	1.182	0.9	0.971	0.798	1.194	0.8
EF < 35%	0.981	0.014	1.217	0.9	5.04	1.66	15.29	0.004**
VITALS_SBP	0.892	0.845	0.942	0.001**	1.013	0.991	1.035	0.2
VITALS_DBP	0.952	0.922	0.982	0.002**	0.982	0.955	1.011	0.2
CREA	1.028	1.014	1.041	0.001**	1.017	1.004	1.029	0.008**
CKMB	1.002	1.001	1.003	0.002**	0.998	0.995	1.001	0.2
PLT	0.977	.967	.986	0.001**	1.004	.999	1.009	0.1

*AOR* adjusted odd ratio, *CI* confidence interval, *p *value calculated depend on logistic regression analysis

^*^P value < 0.05 is significant, **p value < 0.01 is highly significant

When analyzing the overall mortality in the whole study population, we found past history of hypertension, lower LVEF and higher creatinine levels were associated with a greater risk of death; in addition, Post-PPCI–IABP group had significantly worse outcomes when compared with Pre-PPCI–IABP (AOR 4.992, *P* value 0.001) (Table 4).

**Table 4:** Risk assessment of the Risk factors on the death rate the studied patients

Risk factors		Death rate			
		AOR	95.0% C.I		*P* value
			Lower Bound	Upper Bound	
AGE		1.010	0.984	1.037	0.462
SEX		0.933	0.366	2.378	0.885
HTN		1.950	0.993	3.831	0.05*
DM		1.481	0.796	2.758	0.215
VITALS_SpO2		0.908	0.854	0.966	0.002**
RISK_FACTORS_DL		0.800	0.402	1.592	0.525
RISK_FACTORS_CKD		1.1	0.89	1.02	0.999
EF < 35%		4.500	2.022	10.016	0.001**
HB		1.003	0.892	1.127	0.963
CREA TROP		1.007	1.002	1.011	0.003**
CKMB		1.013	0.996	1.031	0.123
PLT		1.001	1.000	1.002	0.062
SYMPTOMS_DURATION STEMI	1.007	0.988	1.027	0.481	0.001**
	0.750	0.353	1.592	0.454	0.999
LMS		1.077	0.488	2.376	0.854
LAD		2.100	1.080	4.085	0.029*
LCX		2.800	1.365	5.742	0.005**
RCA		1.100	0.583	2.075	0.768
MVD		0.533	0.286	0.993	0.047*
COMPLETE_REV		2.121	1.021	4.406	0.044*
Glycoprotein inhibitors IIb/IIIa		1.200	0.627	2.297	0.582
HIGH_VASOPRESSORS		1.540	0.812	2.922	0.186
MECHANICAL VENTILATION		1.091	0.561	2.120	0.797
IABP POST-CATH Vs PRE		4.992	2.579	9.665	0.001**

*AOR* adjusted odd ratio, *CI* confidence interval, *p *value calculated depend on logistic regression analysis

^*^P value < 0.05 is significant, **p value < 0.01 is highly significant

## Discussion

In the present study, we analyze the effect of IABP insertion before and after PPCI in acute myocardial infarction. We found that despite the longer door-to-balloon time with Pre-PPCI–IABP insertion, the in-hospital and 6-month survival was significantly better than that of the Post-PPCI–IABP group.

Hemodynamic instability and CS affect 6–9% of patients presenting with AMI increasing the hospital mortality rate by approximately 50% [14]. In spite of the use of mechanical circulatory support and early revascularization strategies, CS remains the main cause of death in patients with AMI [15].

Currently, IABP is the most used device for mechanical circulatory support in patients with CS related to AMI [16]. Experimental and clinical studies of IABP demonstrated a hemodynamic benefit due to after-load reduction, diastolic augmentation, improvement of coronary and prolonged IABP placement may increase the risk of thrombosis, malignant arrhythmia, heart failure, infection, and pulmonary embolism. Early IABP insertion will result in a faster improvement in coronary perfusion, but it may delay revascularization of the culprit lesion by extending the door-to-balloon time which may increase the perioperative mortality rate. On the other hand, late IABP insertion may delay the positive effect of IABP on coronary perfusion and increase the mortality rate [17].

Reports analyzing the benefits of IABP post-AMI have conflicting results [18]. The SHOCK registry confirmed IABP benefit in reducing in-hospital mortality [19], whereas the IABP-SHOCK II (Intra-aortic Balloon Pump in Cardiogenic Shock II) showed that IABP did not reduce 30-day mortality with no evidence of long-term benefit; however, these results might be affected by the high frequency of crossover (10–30%) between the groups. Additionally, only 13.4% of the IABP were inserted before revascularization which was less than this study (30.9%) [8].

The latest European Society of Cardiology guidelines for myocardial revascularization were largely influenced by the SHOCK II trial results, and have downgraded the recommendation of IABP in treating CS post-AMI to Class IIb, but it did not mention when IABP treatment should start [20].

A recent meta-analysis of 12 randomized trials concluded that IABPs did not reduce the short- or long-term mortality in AMI either with or without CS although the timing of insertion of IABP was not mentioned in many of these trials [21].

In this study patients with Pre-PPCI–IABP suffered more Killip IV, lower systolic blood pressure readings, more prevalence of renal insufficiency, and lower LVEF than the Post-PPCI–IABP. While the Pre-PPCI population has more involvement in left main and three-vessel disease as compared to Post-PPCI–IABP.

Abdel-Wahab et al. analyzed the effect of IABP in 48 patients with AMI and CS and found a better survival rate with Pre-PPCI–IABP than IABP after PPCI [22]. In contrast, a meta-analysis study illustrated that patients who received IABP therapy before PPCI had similar short-term and long-term mortality compared to those who received IABP therapy after PPCI [23]. It is worth mentioning that most of these studies did not report time delays or door-to-balloon time.

In the present study, although patients with Pre-PPCI–IABP had significantly longer door-to-balloon time in comparison with Post-PPCI–IABP, the latter had better overall survival benefit which could be related to early restoration of coronary perfusion with the IABP.

The differences in outcome observed in our study may be explained in part by the early hemodynamic stabilization in Pre-PPCI–IABP, and less prevalence of left main disease and three-vessel diseases. That possibly minimized the need for high doses of inotropes and enabled a higher number of complete revascularizations when compared to Post-PPCI–IABP.

Complete revascularization was achieved in Post-PPCI–IABP more commonly than in the Pre-PPCI–IABP group, which was a protective factor for in-hospital and short/intermediate-term mortality. In agreement with our results, the COMPLETE randomized trial showed 7.8% absolute reduction in cardiovascular death, new MI and revascularization in the complete revascularization group after a 3-year follow-up [24]. On the contrary, the Culprit-Shock trial showed the rate of death from any cause was significantly lower in the culprit-lesion-only PCI group than in the multi-vessel PCI group [25].

In this study, the higher prevalence of low LVEF in Pre-PPCI–IABP LVEF < 30% was a major predictor for mortality of the whole population and was clearly pronounced in Post-PPCI–IABP group, which came in agreement with Brezinov et al. who demonstrated that LVEF is a powerful predictor of 1-year mortality in ACS^26^.

One of the limitations of this study is a single-center non-randomized retrospective trial that is underpowered to reliably detect a mortality difference between the study groups. Moreover, there is no other investigation (secondary endpoints) like recurrent myocardial infarction, stroke and bleeding. Longer follow-up should have been pursued for the detection of long-term mortality and other secondary endpoints.

## Conclusions

This study showed that inserting IABP before PPCI in AMI with hemodynamic instability had a significant survival benefit during the hospital stay that extends to 6 months after hospital discharge. Powered randomized trials are warranted to investigate the relative benefit of the two strategies, that is, IABP inserted before or after PPCI in future.

## Data Availability

The data that support the findings of this study are available from the electronic system of the cardiology department in King Saud medical city, but restrictions apply to the availability of these data, which were used under license for the current study, and so are not publicly available.

## Abbreviations

ACS: Acute coronary syndrome
AMI: Acute myocardial infarction
CS: Cardiogenic shock
IABP: Intra-aortic balloon counter-pulsation
MACE: Major adverse cardiac events
NSTEMI: Non-ST elevation myocardial infarction
PPCI: Primary percutaneous coronary intervention
STEMI: ST elevation myocardial infarction
